# Complete genome sequence of *Thermosediminibacter oceani* type strain (JW/IW-1228P^T^)

**DOI:** 10.4056/sigs.1133078

**Published:** 2010-09-28

**Authors:** Sam Pitluck, Montri Yasawong, Christine Munk, Matt Nolan, Alla Lapidus, Susan Lucas, Tijana Glavina Del Rio, Hope Tice, Jan-Fang Cheng, David Bruce, Chris Detter, Roxanne Tapia, Cliff Han, Lynne Goodwin, Konstantinos Liolios, Natalia Ivanova, Konstantinos Mavromatis, Natalia Mikhailova, Amrita Pati, Amy Chen, Krishna Palaniappan, Miriam Land, Loren Hauser, Yun-Juan Chang, Cynthia D. Jeffries, Manfred Rohde, Stefan Spring, Johannes Sikorski, Markus Göker, Tanja Woyke, James Bristow, Jonathan A. Eisen, Victor Markowitz, Philip Hugenholtz, Nikos C. Kyrpides, Hans-Peter Klenk

**Affiliations:** 1 DOE Joint Genome Institute, Walnut Creek, California, USA; 2 HZI – Helmholtz Centre for Infection Research, Braunschweig, Germany; 3 Los Alamos National Laboratory, Bioscience Division, Los Alamos, New Mexico, USA; 4 Biological Data Management and Technology Center, Lawrence Berkeley National Laboratory, Berkeley, California, USA; 5 Oak Ridge National Laboratory, Oak Ridge, Tennessee, USA; 6 DSMZ - German Collection of Microorganisms and Cell Cultures GmbH, Braunschweig, Germany; 7 University of California Davis Genome Center, Davis, California, USA

**Keywords:** chemoorganotroph, anaerobe, thermophile, barophile, upwelling system, core sample, deep sea sediment, *Thermoanaerobacterales*, *Firmicutes*, GEBA

## Abstract

*Thermosediminibacter oceani* (Lee *et al*. 2006) is the type species of the genus *Thermosediminibacter* in the family *Thermoanaerobacteraceae*. The anaerobic, barophilic, chemoorganotrophic thermophile is characterized by straight to curved Gram-negative rods. The strain described in this study was isolated from a core sample of deep sea sediments of the Peruvian high productivity upwelling system. This is the first completed genome sequence of a member of the genus *Thermosediminibacter* and the seventh genome sequence in the family *Thermoanaerobacteraceae*. The 2,280,035 bp long genome with its 2,285 protein-coding and 63 RNA genes is a part of the *** G****enomic* *** E****ncyclopedia of* *** B****acteria and* *** A****rchaea * project.

## Introduction

Strain JW/IW-1228P^T^ (= DSM 16646 = ATCC BAA-1034) is the type strain of *Thermosediminibacter oceani*, which is the type species of the genus *Thermosediminibacter* [1], one out of nineteen genera in the family *Thermoanaerobacteraceae* [2-4]. The generic name derives from the Greek word ‘*thermos*’ meaning ‘hot’, the Latin word ‘*sediment*’ and the Latin word ‘*bacter*’ meaning ‘a rod or staff’, referring to its origin and growth temperature [1]. The species epithet is also derived from the Latin word ‘*oceani*’ meaning ‘of an ocean’, referring to its origin from the ocean [1]. Strain JW/IW-1228P^T^ was described in 2005 by Lee as *T. oceani* [1] and validly published in 2006 [5]. Strain JW/IW-1228P^T^ was isolated from a core sediment sample (core 201-1228E-1H-1) at 136-143 cm below the seafloor. The core sample was obtained from the outer shelf edge of the Peruvian high productivity upwelling system. The sea floor there was located at 252 m below the sea level with 12°C mud line temperature. Strain JW/IW-1228P^T^ is of particular interest because it is able to ferment a significant number of polysaccharides [1]. Moreover, the strain JW/IW-1228P^T^ is able to use thiosulfate, elemental sulfur and MnO_2_ as electron acceptors for growth. The only other species in the genus *Thermosediminibacter* is *T. litoriperuensis*, the type strain of which was isolated from the Peru Trench at 5,086 m below sea level with a mud-line temperature of 2°C [1]. Here we present a summary classification and a set of features for *T. oceani* JW/IW-1228P^T^, together with the description of the complete genomic sequencing and annotation.

## Classification and features

The 16S rRNA gene sequence of JW/IW-1228P^T^ is 98.4% identical to that of *T. litoriperuensis* JW/YJL-1230-2^T^, the type strain of the only other described species with a validly published name in the genus. The sequence similarities between strain JW/IW-1228P^T^ and the type strains of the members of the genera *Fervidicola* and *Caldanaerovirga* are 94.4%, with the closest sequence match being that with *F. ferrireducens* and *C. acetigignens* [6]. Three significantly similar 16S rRNA gene sequences are known from uncultured clones of *Thermovenabulum* sp. from GenBank [7]: B5_otu10 (96%, DQ097675), B14_otu11 (95%, DQ097676) and B8_otu12 (95%, DQ097677), all from the Kongdian bed of the Dagang oil field (Hebei province, China) [7,8]. No phylotypes from environmental screening or genomic surveys could be linked to the species *T. oceani* or even the genus *Thermosediminibacter*, indicating a rather rare occurrence of these in the habitats screened so far (as of July 2010).

**Figure 1:**
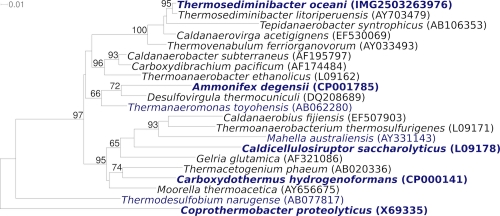
Phylogenetic tree highlighting the position of *T. oceani* JW/IW-1228P^T^ relative to the type strains of the other species within the family *Thermoanaerobacteraceae*. The trees were inferred from 1,316 aligned characters [9,10] of the 16S rRNA gene sequence under the maximum likelihood criterion [11] and rooted in accordance with the current taxonomy [12]. The branches are scaled in terms of the expected number of substitutions per site. Numbers above branches are support values from 850 bootstrap replicates [13] if larger than 60%. Lineages with type strain genome sequencing projects registered in GOLD [14] are shown in blue, published genomes in bold [32,33,CP001785,CP001145].

Figure 1 shows the phylogenetic neighborhood of *T. oceani* JW/IW-1228P^T^ in a 16S rRNA based tree. The sequences of the three 16S rRNA gene copies in the genome differ from each other by up to one nucleotide and differ by only one nucleotide from the previously published sequence (AY703478).

**Figure 2:**
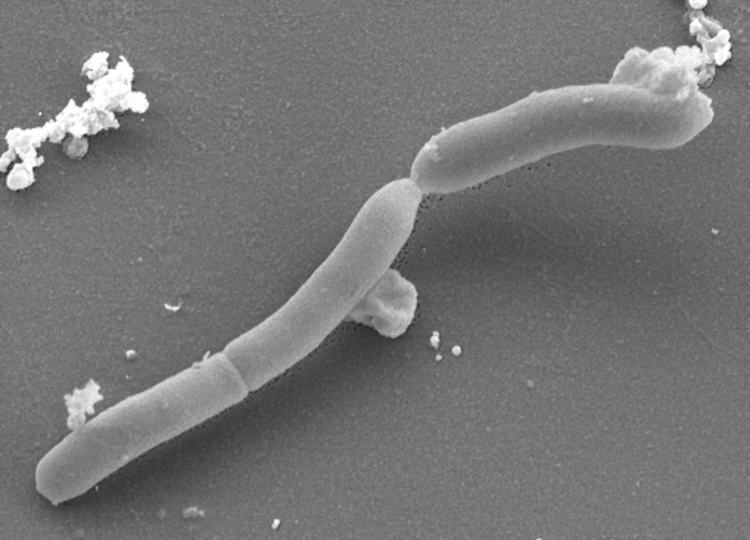
Scanning electron micrograph of *T. oceani* JW/IW-1228P ^T^

The cells of strain JW/IW-1228P^T^ are straight to curved rods which occur singly, in pairs or in chains (Table 1 and Figure 2). They are between 0.2-0.7µm in diameter and 1.5-16 µm in length. In the late-exponential or stationary phase of growth the cells are swollen and subsequently form L-shaped autoplasts [1]. Strain JW/IW-1228P^T^ is Gram-negative, although *Thermosediminibacter* belongs to the Gram-positive *Bacillus*-*Clostridium* subphylum [1]. The cells tend towards elongation and to form aggregates during growth. Motility has not been reported although flagella are observed on the cells (not visible in Figure 2), however, the cells are able to tumble [1], which might imply an impaired flagellar function. Strain JW/IW-1228P^T^ is thermophilic and grows optimally at 68°C; the temperature range for growth is 52-76°C. The optimum pH^25°C^ for growth is 7.5, with a range for growth at 6.3-9.3. The optimum salinity for growth is 1% (w/v), with a salinity range of 0-6% (w/v) [1]. Yeast extract is required for growth. The growth of strain JW/IW-1228P^T^ is not observed on H_2_/CO_2_ (80:20, v/v) [1]. The strain produces *α*-glucosidase [22]. The carbon and energy sources used by JW/IW-1228P^T^ include beef extract, casamino acids, cellobiose, fructose, galactose, glucose, inositol, lactate, maltose, mannose, pyruvate, raffinose, sorbitol, sucrose, trehalose, tryptone and xylose when 0.02% w/v of yeast extract is present in growth medium [1]. The fermentation product from glucose is acetate and occasionally trace amounts of propionate, isobutyrate and isovalerate. Acetate is a major product [1]. Strain JW/IW-1228P^T^ does not utilize xylitol [22]. It is able to use thiosulfate, elemental sulfur and MnO_2_ as electron acceptors for growth. There is no indication that JW/IW-1228P^T^ is able to grow chemolithoautotrophically; it does not reduce sulfate or Fe(III) [1].

**Table 1:** Classification and general features of *T. oceani* JW/IW-1228P ^T^ according to the MIGS recommendations [15].

**MIGS ID**	**Property**	**Term**	**Evidence code**
	Current classification	Domain *Bacteria*	TAS [16]
		Phylum *Firmicutes*	TAS [17,18]
		Class *Clostridia*	TAS [2,19]
		Order *Thermoanaerobacterales*	TAS [2,19,20]
		Family *Thermoanaerobacteraceae*	TAS [2-4]
		Genus *Thermosediminibacter*	TAS [1,5]
		Species *Thermosediminibacter oceani*	TAS [1,5]
		Type strain JW/IW-1228P	TAS [1,5]
	Gram stain	negative	TAS [1]
	Cell shape	straight to curved rods, 0.2-0.7 ×1.5-16 µm. cells tend to elongate and form aggregates.	TAS [1]
	Motility	no motility, but tumbling (flagella observed)	TAS [1]
	Sporulation	not observed	TAS [1]
	Temperature range	52–76°C	TAS [1]
	Optimum temperature	68°C	TAS [1]
	Salinity	0-6% w/v NaCl (optimum at 1%)	TAS [1]
MIGS-22	Oxygen requirement	anaerobic	TAS [1]
	Carbon source	carbohydrates	TAS [1]
	Energy source	chemoorganotroph	TAS [1]
MIGS-6	Habitat	ocean subsurface sediments	TAS [1]
MIGS-15	Biotic relationship	free-living	NAS
MIGS-14	Pathogenicity	none	NAS
	Biosafety level	1	TAS [21]
	Isolation	core sample from deep sea sediment	TAS [1]
MIGS-4	Geographic location	subseafloor, outer shelf edge of the Peruvian high productivity upwelling system, Peru	TAS [1]
MIGS-5	Sample collection time	2002	NAS
MIGS-4.1	Latitude	approx. S11° 11' 23"	TAS [22]
MIGS-4.2	Longitude	approx. W79° 4' 33"	TAS [22]
MIGS-4.3	Depth	136-143 cm below seafloor	TAS [1]
MIGS-4.4	Altitude	252 m below sea level	TAS [1]

Evidence codes - IDA: Inferred from Direct Assay (first time in publication); TAS: Traceable Author Statement (i.e., a direct report exists in the literature); NAS: Non-traceable Author Statement (i.e., not directly observed for the living, isolated sample, but based on a generally accepted property for the species, or anecdotal evidence). These evidence codes are from of the Gene Ontology project [23]. If the evidence code is IDA, then the property was directly observed by one of the authors or an expert mentioned in the acknowledgements

### Chemotaxonomy

The peptidoglycan structure of strain JW/IW-1228P^T^ is still unknown. The phospholipid fatty acid composition of strain JW/IW-1228P^T^ consists of branched and straight chain saturated acids: iso-C_15:0_ (56.2%), iso-C_17:0_ (9.6%), C_16:0_ (7.5%), anteiso-C_15:0_ (6.7%), C_16:1_ω9c (5.6%), C_15:0_ (5.0%), C_18:1_ ω 9c (3.3%) and iso-C_16:0_ (1.9%) [1].

## Genome sequencing and annotation

### Genome project history

This organism was selected for sequencing on the basis of its phylogenetic position [24], and is part of the *** G****enomic* *** E****ncyclopedia of* *** B****acteria and* *** A****rchaea * project [25]. The genome project is deposited in the Genome OnLine Database [14] and the complete genome sequence is deposited in GenBank. Sequencing, finishing and annotation were performed by the DOE Joint Genome Institute (JGI). A summary of the project information is shown in Table 2.

**Table 2:** Genome sequencing project information

**MIGS ID**	**Property**	**Term**
MIGS-31	Finishing quality	Finished
MIGS-28	Libraries used	Tree genomic libraries: one Sanger 8 kb pMCL200 library, one 454 pyrosequence standard library and one Illumina standard library
MIGS-29	Sequencing platforms	ABI3730, Illumina GAii, 454 GS FLX Titanium
MIGS-31.2	Sequencing coverage	5.3× Sanger; 34.3× Illumina, 25.4× pyrosequence
MIGS-30	Assemblers	Newbler version 2.0.0-PostRelease- 07/15/2008, Velvet, phrap
MIGS-32	Gene calling method	Prodigal 1.4, GenePRIMP
	INSDC ID	CP002131
	Genbank Date of Release	August 5, 2010
	GOLD ID	Gc01361
	NCBI project ID	30983
	Database: IMG-GEBA	2503242007
MIGS-13	Source material identifier	DSM 16646
	Project relevance	Tree of Life, GEBA

### Growth conditions and DNA isolation

*T. oceani* JW/IW-1228P^T^, DSM 16646, was grown anaerobically in DSMZ medium 664 (*Thermotoga elfii* medium) [26] at 68°C. DNA was isolated from 0.5-1 g of cell paste using Qiagen Genomic 500 DNA Kit (Qiagen, Hilden, Germany) following the standard protocol as recommended by the manufacturer, with modification st/LALMP for cell lysis as described in Wu *et al*. [25].

### Genome sequencing and assembly

The genome was sequenced using a combination of Sanger, Illumina and 454 sequencing platforms. All general aspects of library construction and sequencing can be found at the JGI website (http://www.jgi.doe.gov/). Pyrosequencing reads were assembled using the Newbler assembler version 2.0.0-PostRelease-07/15/2008 (Roche). The initial Newbler assembly consisted of 83 contigs in 32 scaffolds which was converted into a phrap assembly by making fake reads from the consensus. Illumina GAii sequencing data was assembled with Velvet [27] and the consensus sequences were shredded into 1.5 kb overlapped fake reads and assembled together with the 454 data. Draft assemblies were based on 166.4 Mb 454 draft data and all of the 454 paired end data. Newbler parameters are -consed -a 50 -l 350 -g -m -ml 20. The Phred/Phrap/Consed software package (www.phrap.com) was used for sequence assembly and quality assessment in the following finishing process. After the shotgun stage, reads were assembled with parallel phrap (High Performance Software, LLC). Possible mis-assemblies were corrected with Dupfinisher **[**28**],** or sequencing cloned bridging PCR fragments with subcloning. Gaps between contigs were closed by editing in Consed, by PCR and by Bubble PCR primer walks (J.-F.Chang, unpublished). A total of 625 additional reactions and two shatter libraries were necessary to close gaps and to raise the quality of the finished sequence. Illumina data was used to correct potential base errors and increase consensus quality using a software Polisher developed at JGI [29]. The error rate of the completed genome sequence is less than 1 in 100,000. Together, the combination of the Sanger, Illumina and 454 sequencing platforms provided 65.0 ×coverage of the genome.

### Genome annotation

Genes were identified using Prodigal [30] as part of the Oak Ridge National Laboratory genome annotation pipeline, followed by a round of manual curation using the JGI GenePRIMP pipeline [31]. The predicted CDSs were translated and used to search the National Center for Biotechnology Information (NCBI) nonredundant database, UniProt, TIGRFam, Pfam, PRIAM, KEGG, COG, and InterPro databases. Additional gene prediction analysis and functional annotation was performed within the Integrated Microbial Genomes - Expert Review (IMG-ER) platform [32].

## Genome properties

**Figure 3:**
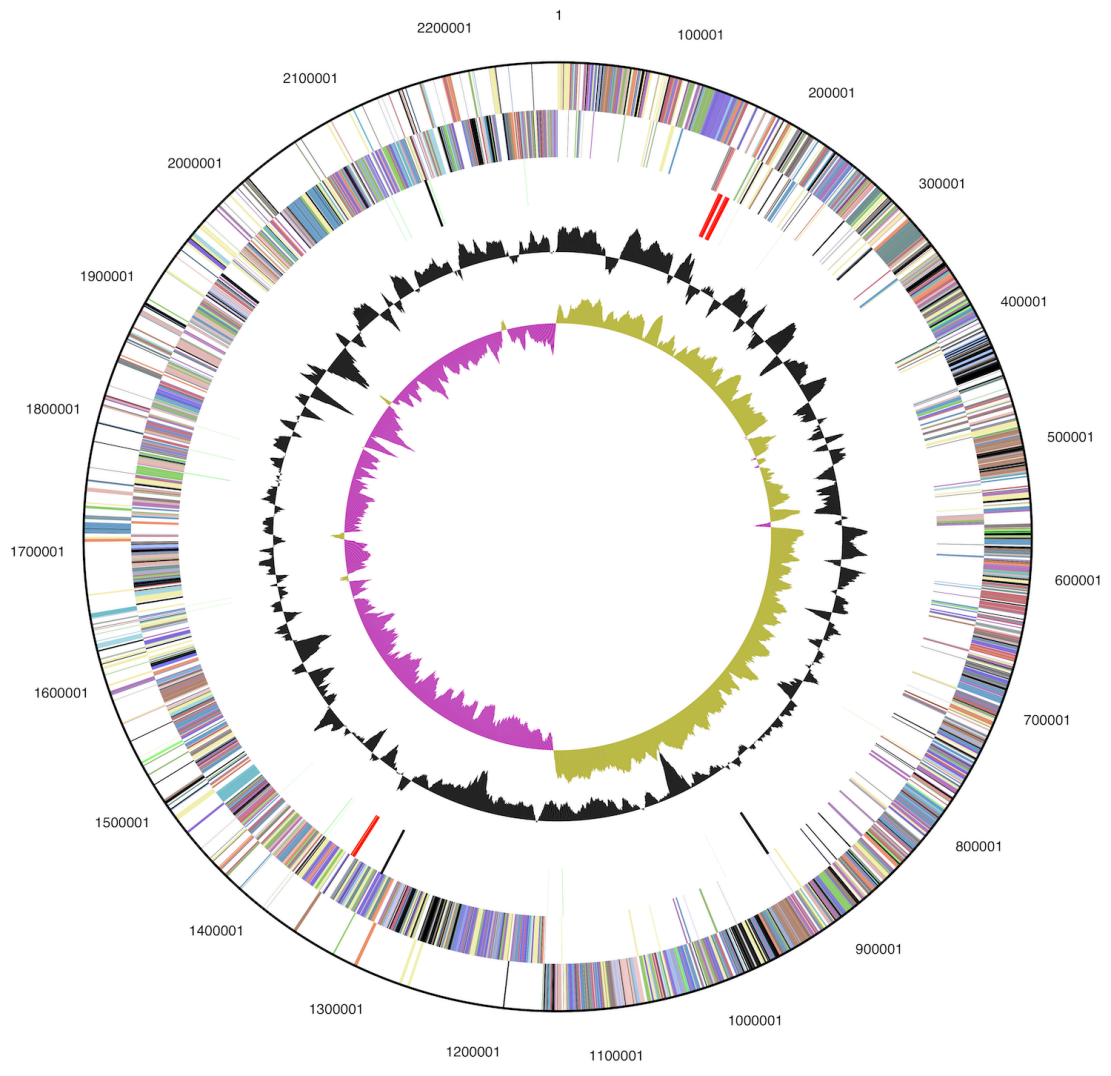
Graphical circular map of the genome. From outside to the center: Genes on forward strand (color by COG categories), Genes on reverse strand (color by COG categories), RNA genes (tRNAs green, rRNAs red, other RNAs black), GC content, GC skew.

The genome consists of a 2,280,035 bp long chromosome with a 468% GC content (Table 3 and Figure 3). Of the 2,348 genes predicted, 2,285 were protein-coding genes, and 63 RNAs; eighty eight pseudogenes were also identified. The majority of the protein-coding genes (73.3%) were assigned with a putative function while the remaining ones were annotated as hypothetical proteins. The distribution of genes into COGs functional categories is presented in Table 4.

**Table 3:** Genome Statistics

**Attribute**	**Value**	**% of Total**
Genome size (bp)	2,280,035	100.00%
DNA coding region (bp)	1,991,971	87.37%
DNA G+C content (bp)	1,067,515	46.82%
Number of replicons	1	
Extrachromosomal elements	0	
Total genes	2,348	100.00%
RNA genes	63	2.68%
rRNA operons	3	
Protein-coding genes	2,285	97.32%
Pseudo genes	88	3.75%
Genes with function prediction	1,722	73.34%
Genes in paralog clusters	366	15.59%
Genes assigned to COGs	1,751	74.57%
Genes assigned Pfam domains	1,925	81.98%
Genes with signal peptides	280	11.93%
Genes with transmembrane helices	563	23.98%
CRISPR repeats	5	

**Table 4:** Number of genes associated with the general COG functional categories

**Code**	**Value**	**%age**	**Description**
J	140	7.3	Translation, ribosomal structure and biogenesis
A	0	0.0	RNA processing and modification
K	122	6.4	Transcription
L	191	10.0	Replication, recombination and repair
B	1	0.1	Chromatin structure and dynamics
D	35	1.8	Cell cycle control, cell division, chromosome partitioning
Y	0	0.0	Nuclear structure
V	28	1.5	Defense mechanisms
T	96	5.0	Signal transduction mechanisms
M	107	5.6	Cell wall/membrane/envelope biogenesis
N	58	3.0	Cell motility
Z	0	0.0	Cytoskeleton
W	0	0.0	Extracellular structures
U	49	2.6	Intracellular trafficking and secretion, and vesicular transport
O	58	3.0	Posttranslational modification, protein turnover, chaperones
C	142	7.4	Energy production and conversion
G	117	6.1	Carbohydrate transport and metabolism
E	147	7.7	Amino acid transport and metabolism
F	51	2.7	Nucleotide transport and metabolism
H	94	4.9	Coenzyme transport and metabolism
I	33	1.7	Lipid transport and metabolism
P	85	4.5	Inorganic ion transport and metabolism
Q	25	1.3	Secondary metabolites biosynthesis, transport and catabolism
R	171	9.0	General function prediction only
S	158	8.3	Function unknown
-	597	25.4	Not in COGs
